# Tirzepatide and reduced risk of pulmonary embolism and deep vein thrombosis: a multicenter U.S. cohort study

**DOI:** 10.3389/fendo.2026.1885961

**Published:** 2026-07-01

**Authors:** Fredy Kahkedjian, Jaffer Shah, Firas Kreidieh

**Affiliations:** 1 Faculty of Medicine, American University of Beirut, Beirut, Lebanon; 2 Department of Ophthalmology, Weill Cornell Medicine, New York, NY, United States; 3 Department of Internal Medicine, Division of Hematology-Oncology, American University of Beirut Medical Center, Beirut, Lebanon

**Keywords:** deep vein thrombosis, GLP-1 receptor agonist, obesity, pulmonary embolism, real-world study, thrombosis, tirzepatide, TriNetX

## Abstract

**Background:**

Tirzepatide is a dual glucose-dependent insulinotropic polypeptide and glucagon-like peptide-1 receptor agonist used in patients with type 2 diabetes and obesity. Although tirzepatide improves metabolic and cardiovascular risk factors, its association with venous thromboembolism, including pulmonary embolism and deep vein thrombosis, remains insufficiently characterized in real-world populations.

**Methods:**

We conducted a population-based retrospective cohort study using the TriNetX US Collaborative Network. Adults with type 2 diabetes and overweight or obesity who initiated tirzepatide were propensity score matched to patients receiving lifestyle intervention alone with no exposure to weight-loss medications. Outcomes included incident pulmonary embolism, deep vein thrombosis, and superficial vein thrombosis occurring between 30 days and 12 months after index. Additional analyses included a 90-day landmark sensitivity analysis and an active comparator analysis comparing tirzepatide with semaglutide.

**Results:**

After propensity score matching for demographic, metabolic, and clinical covariates, 235, 200 patients were included in the primary analysis (117, 600 per cohort). Tirzepatide use was associated with a significantly lower 12-month risk of pulmonary embolism compared with lifestyle intervention alone (RR, 0.215; 95% CI, 0.185–0.250; HR, 0.258; 95% CI, 0.222–0.299; log-rank *P* < 0.001). Similar reductions were observed for deep vein thrombosis (RR, 0.303; 95% CI, 0.270–0.340; HR, 0.361; 95% CI, 0.322–0.406; log-rank *P* < 0.001). No statistically significant difference was observed for superficial vein thrombosis (RR, 0.716; 95% CI, 0.484–1.060; HR, 0.868; 95% CI, 0.586–1.286; log-rank P = 0.480). Findings for pulmonary embolism and deep vein thrombosis remained significant in the 90-day landmark analysis. In the semaglutide comparator analysis, tirzepatide was associated with a significantly lower risk of deep vein thrombosis, while the pulmonary embolism association was significant by risk-ratio analysis but not by Cox regression.

**Conclusions:**

In this large multicenter cohort study, tirzepatide use was associated with a lower risk of pulmonary embolism and deep vein thrombosis compared with lifestyle intervention alone. These findings suggest a potential association between tirzepatide use and lower observed rates of thromboembolic events in patients with diabetes and obesity.

## 1 Introduction

Venous thromboembolism (VTE), encompassing pulmonary embolism (PE) and deep vein thrombosis (DVT), remain a major cause of morbidity and mortality worldwide, with an estimated annual incidence of approximately 1 to 2 events per 1, 000 persons in the general population (1). Patients with obesity and type 2 diabetes mellitus (T2DM) are at elevated thrombotic risk because of a prothrombotic milieu characterized by endothelial dysfunction, chronic inflammation, platelet activation, and impaired fibrinolysis (2). As the incidence of obesity and diabetes continues to rise globally, identifying therapeutic options that may mitigate thromboembolic risk ensues.

Incretins are a group of hormones produced by the gastrointestinal tract to enhance insulin secretion by pancreatic beta cells. Tirzepatide is a dual incretin agonist that activates both glucose-dependent insulinotropic polypeptide (GIP) and glucagon-like peptide-1 (GLP-1) receptors. Tirzepatide demonstrates substantial efficacy in improving glycemic control and reducing body weight in patients with type 2 diabetes, as shown by a meta-analysis of four randomized controlled trials involving 2783 patients (3). These data suggest its potential role for lowering thromboembolic risk through metabolic improvements. However, while incretin-based therapies have been extensively studied in relation to glycemic and cardiovascular outcomes, the association between tirzepatide and venous thromboembolic events remains poorly described.

Recent evidence has suggested that GLP-1 receptor agonists may have neutral to modestly favorable effects on VTE risk, and observational work has linked GLP-1 receptor agonist use with lower 1-year VTE risk in patients with T2DM (4). Whether tirzepatide specifically, with its dual GIP/GLP-1 mechanism and greater metabolic efficacy, confers similar or greater benefit remains unclear.

Although tirzepatide has demonstrated substantial metabolic and cardiovascular benefits, emerging case reports have raised concerns regarding potential thromboembolic events following its initiation (5). These reports describe instances of pulmonary embolism occurring shortly after treatment initiation, including in patients with minimal or no identifiable risk factors (6). However, the relationship between tirzepatide and venous thromboembolism remains uncertain, highlighting the need for large-scale real-world studies to better characterize this association. Unlike prior general drug class-level analyses of GLP-1 receptor agonists (7), our study specifically evaluates tirzepatide and examines its association with pulmonary embolism, deep vein thrombosis, and superficial vein thrombosis using lifestyle intervention, 90-day landmark, and semaglutide active-comparator analyses.

## 2 Methods

This was a retrospective cohort study that followed the Strengthening the Reporting of Observational Studies in Epidemiology (STROBE) reporting guideline (8).

### 2.1 Data source

This population-based retrospective cohort study used the TriNetX US Collaborative Network, a federated electronic health record database comprising over 120 million patients across more than 70 healthcare organizations in the United States. According to the analysis export, the present query included 67 contributing healthcare organizations, all of which responded to the query. Data analysis was conducted on March 14, 2026. The database is compliant with the Health Insurance Portability and Accountability Act (HIPAA) and certified under ISO 27001:2013 standards. As all data are de-identified and used for population-level analyses, this study was considered exempt from institutional review board oversight and informed consent requirements. The study adhered to the principles of the Declaration of Helsinki.

### 2.2 Study population

We included adult patients with type 2 diabetes mellitus and either a diagnosis of overweight or obesity or a most recent body mass index (BMI) of at least 27 kg/m². Type 2 diabetes mellitus was used as an inclusion criterion and was therefore not included as a propensity score matching variable.

The tirzepatide cohort consisted of patients prescribed tirzepatide with no prior exposure to other weight-loss medications included in the query definition, including phentermine, semaglutide, liraglutide, setmelanotide, orlistat, naltrexone, and bupropion. Patients with a history of pulmonary embolism, deep vein thrombosis, or superficial vein thrombosis before the index date were excluded.

The comparison cohort included patients receiving lifestyle intervention alone, defined by documentation of dietary counseling, exercise counseling, face-to-face behavioral counseling for obesity, or medical nutrition therapy, with no previous lifestyle intervention, no prior history of pulmonary embolism, deep vein thrombosis, or superficial vein thrombosis, and no exposure to weight-loss medications included in the query definition.

Both cohorts excluded patients with prior bariatric surgery.

Detailed coding definitions used for cohort construction and outcome ascertainment are provided in the **Supplementary Materials**.

### 2.3 Index date and follow-up

The index date was defined as the first qualifying tirzepatide prescription in the exposure cohort and the first qualifying lifestyle intervention in the comparator cohort. Outcomes were assessed from 30 days to 365 days after the index date to reduce early surveillance and protopathic bias. As a sensitivity analysis, a 90-day landmark analysis was performed, with outcome assessment beginning 90 days after the index date and continuing through 365 days.

### 2.4 Outcomes

The primary outcome was incident pulmonary embolism. Secondary outcomes included acute deep vein thrombosis of the lower extremities and superficial thrombophlebitis of superficial lower-extremity vessels.

Outcomes were identified using electronic health record diagnostic coding within the TriNetX platform. Patients with the relevant outcome before the beginning of the outcome window were excluded from the corresponding outcome-specific analysis.

Because this was a federated electronic health record study, imaging confirmation, venous segment localization, thrombus burden, and adjudicated clinical validation were not available.

### 2.5 Covariates and propensity score matching

Propensity score matching was performed in a 1:1 fashion. Matching variables included demographic characteristics, clinical comorbidities, prior healthcare utilization, systemic contraceptive use, and laboratory variables. Specifically, patients were matched on age, sex, race, ethnicity, hypertension, insulin use, atherosclerotic coronary disease, hypercholesterolemia, disorders of lipoprotein metabolism, neoplasms, ischemic heart disease, chronic kidney disease, nicotine dependence, surgery, hospital inpatient and observation care services, BMI, hemoglobin A1c, and total cholesterol.

These covariates were selected to improve comparability between cohorts and to account for measured differences in cardiometabolic risk, obesity severity, malignancy-related diagnoses, healthcare utilization, surgical exposure, smoking-related risk, anti-coagulant/anti-platelet status, and hormonal contraceptive exposure. More details about the covariates are available in **Table 1**; **Supplementary Materials**.

**Table 1:** Baseline characteristics of propensity score-matched cohorts.

Variable	Tirzepatide (n=117, 600)	Lifestyle intervention (n=117, 600)	SMD
Age, years	57.6 ± 13.1	58.1 ± 15.8	0.037
Female sex	53.6%	53.6%	<0.001
BMI, kg/m²	37.3 ± 7.5	36.5 ± 7.8	0.101
White	66.3%	66.7%	0.008
Black or African American	19.9%	19.4%	0.011
Hispanic or Latino	7.2%	6.9%	0.013
Hemoglobin A1c, %	7.5 ± 1.9	7.5 ± 2.0	0.015
Hypertension	68.4%	66.7%	0.036
Ischemic heart disease	21.0%	20.4%	0.016
Cerebral infarction	4.3%	4.2%	0.006
Heart failure	11.2%	10.8%	0.013
Chronic kidney disease	15.4%	14.8%	0.016
Nicotine dependence	12.7%	12.2%	0.016
Anticoagulant/antiplatelet use	9.1%	8.8%	0.010
Primary thrombophilia	0.7%	0.7%	<0.001
Varicose veins	3.4%	3.3%	0.006
Hormone replacement therapy	3.6%	3.5%	0.004
Pregnancy	1.5%	1.4%	0.009
Surgery	71.2%	68.4%	0.060
Hospitalization	18.5%	17.8%	0.018
Chemotherapy exposure	8.3%	8.1%	0.006
Obstructive sleep apnea	26.2%	25.6%	0.013

Values are presented as mean ± SD or percentage (%). Standardized mean differences (SMDs) were used to assess covariate balance after propensity score matching. An SMD <0.10 is generally considered indicative of acceptable balance. Baseline covariates were well balanced after matching, with all SMDs ≤0.10 except BMI (SMD = 0.101).

After matching, each cohort included 117, 600 patients. Matching substantially improved baseline balance across measured covariates, with near-complete overlap of propensity score distributions after matching.

### 2.6 Statistical analysis

Risk ratios (RRs), odds ratios (ORs), and risk differences with corresponding 95% confidence intervals (CIs) were calculated to compare outcomes between tirzepatide and lifestyle intervention cohorts. Time-to-event analyses were performed using Kaplan–Meier methods, with comparisons assessed using the log-rank test. Hazard ratios (HRs) and 95% CIs were derived using Cox proportional hazards models.

Propensity score matching was performed using the TriNetX nearest-neighbor matching algorithm with a caliper of 0.1 pooled standard deviations; and missing data were handled according to TriNetX platform procedures.

To account for differences in follow-up duration between cohorts, incidence rates per 1, 000 person-years were additionally calculated. Sensitivity analyses included a 90-day landmark analysis and an active-comparator analysis in which tirzepatide users were propensity score matched to semaglutide users.

Two-sided *P* values <0.05 were considered statistically significant. All analyses were performed within the TriNetX Analytics Platform.

## 3 Results

We identified 128, 618 patients in the tirzepatide cohort and 327, 855 patients in the lifestyle intervention cohort before matching. Among patients eligible for matching, 128, 618 tirzepatide-treated patients and 308, 895 lifestyle-intervention patients entered the propensity score matching procedure. After 1:1 matching, both cohorts included 117, 600 patients.

Before matching, the tirzepatide cohort had higher mean age and had higher BMI and hemoglobin A1c levels, with additional demographic and clinical differences between cohorts. After matching, baseline balance improved across measured covariates (**Table 1**). Variables specifically relevant to venous thromboembolism risk, including anticoagulant/antiplatelet use (9.1% vs 8.8%), primary thrombophilia (0.7% vs 0.7%), varicose veins (3.4% vs 3.3%), hormone replacement therapy (3.6% vs 3.5%), and pregnancy (1.5% vs 1.4%), were also well balanced between cohorts.

Mean follow-up after matching was 255 ± 132 days in the tirzepatide cohort and 323 ± 99 days in the lifestyle intervention cohort. The corresponding median follow-up was 347 days and 365 days, respectively.

### 3.1 Primary outcome: pulmonary embolism

**Figure 1:**
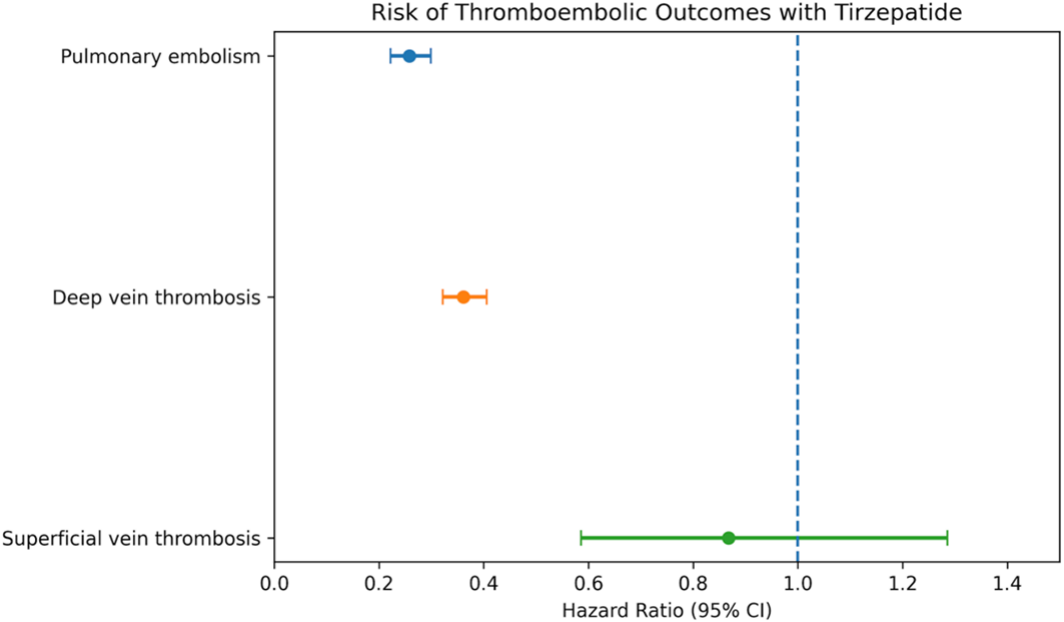
Hazard ratios for thromboembolic outcomes associated with tirzepatide. Forest plot showing hazard ratios (HRs) and 95% confidence intervals (CIs) for pulmonary embolism, deep vein thrombosis, and superficial vein thrombosis comparing tirzepatide with lifestyle intervention. The vertical dashed line represents the null value (HR = 1.0). Estimates to the left of the line indicate lower risk with tirzepatide.

At 12 months, tirzepatide use was associated with a significantly lower risk of pulmonary embolism compared with lifestyle intervention alone (**Table 2**). Pulmonary embolism occurred in 207 of 117, 577 patients (0.18%) in the tirzepatide cohort and 960 of 117, 449 patients (0.82%) in the lifestyle cohort, corresponding to an RR of 0.215 (95% CI, 0.185–0.250) and an OR of 0.214 (95% CI, 0.184–0.249). Kaplan–Meier analysis demonstrated a significantly lower cumulative incidence of pulmonary embolism in the tirzepatide group (log-rank χ² = 362.856; P <0.0001), with an HR of 0.258 (95% CI, 0.222–0.299) (**Figure 1**).

**Table 2:** Risk of thromboembolic outcomes within 12 months.

Outcome	Cohort	Patients in cohort	Events (%)	RR (95% CI)	HR (95% CI)	P value
Pulmonary embolism	Tirzepatide	117, 577	207 (0.18%)	0.215 (0.185–0.250)	0.258 (0.222–0.299)	<0.001
	Lifestyle	117, 449	960 (0.82%)	Reference	Reference	—
Deep vein thrombosis	Tirzepatide	114, 979	381 (0.33%)	0.303 (0.270–0.340)	0.361 (0.322–0.406)	<0.001
	Lifestyle	114, 270	1, 249 (1.09%)	Reference	Reference	—
Superficial vein thrombosis	Tirzepatide	117, 270	43 (0.04%)	0.716 (0.484–1.060)	0.868 (0.586–1.286)	0.480
	Lifestyle	117, 225	60 (0.05%)	Reference	Reference	—

Outcome events were assessed between 30 and 365 days after the index date. Risk ratios (RRs) and hazard ratios (HRs) with 95% confidence intervals (CIs) compare tirzepatide with lifestyle intervention (reference). HRs were derived from Cox proportional hazards models. P values reflect two-sided testing.

When accounting for follow-up duration, pulmonary embolism incidence rates were 2.52 and 9.25 per 1, 000 person-years in the tirzepatide and lifestyle intervention cohorts, respectively (**Table 3**).

**Table 3:** Incidence rates of thromboembolic outcomes.

Outcome	Tirzepatide (per 1, 000 person-years)	Lifestyle (per 1, 000 person-years)
Pulmonary embolism	2.52	9.25
Deep vein thrombosis	4.74	12.37
Superficial vein thrombosis	0.52	0.58

Incidence rates were estimated using event counts divided by total observed person-time derived from cohort-level mean follow-up duration.

### 3.2 Secondary outcomes

Tirzepatide use was also associated with a significantly lower risk of deep vein thrombosis. Events occurred in 381 of 114, 979 patients (0.33%) in the tirzepatide cohort and 1, 249 of 114, 270 patients (1.09%) in the lifestyle cohort, yielding an RR of 0.303 (95% CI, 0.270–0.340) and an HR of 0.361 (95% CI, 0.322–0.406). The corresponding incidence rates were 4.74 and 12.37 per 1, 000 person-years in the tirzepatide and lifestyle intervention cohorts, respectively.

No statistically significant difference was observed for superficial vein thrombosis. Events occurred in 43 of 117, 270 patients (0.04%) in the tirzepatide cohort and 60 of 117, 225 patients (0.05%) in the lifestyle cohort, corresponding to an RR of 0.716 (95% CI, 0.484–1.060). Kaplan–Meier analysis similarly showed no significant difference (HR, 0.868; 95% CI, 0.586–1.286; log-rank P = 0.480). The corresponding incidence rates were 0.52 and 0.58 per 1, 000 person-years, respectively.

In the 90-day landmark sensitivity analysis, the associations for pulmonary embolism and deep vein thrombosis remained directionally consistent and statistically significant. Tirzepatide remained associated with a lower hazard of pulmonary embolism (HR, 0.324; 95% CI, 0.268–0.392; P <0.001) and deep vein thrombosis (HR, 0.436; 95% CI, 0.375–0.506; P <0.001). No significant association was observed for superficial vein thrombosis (HR, 0.854; 95% CI, 0.505–1.442; P = 0.553) (**Table 4**).

**Table 4:** Ninety-day landmark sensitivity analysis.

Outcome	RR (95% CI)	HR (95% CI)	P value
Pulmonary embolism	0.246 (0.203–0.297)	0.324 (0.268–0.392)	<0.001
Deep vein thrombosis	0.330 (0.284–0.383)	0.436 (0.375–0.506)	<0.001
Superficial vein thrombosis	0.639 (0.378–1.078)	0.854 (0.505–1.442)	0.553

In the active-comparator analysis against semaglutide, tirzepatide was associated with a lower cumulative risk of pulmonary embolism by risk-ratio analysis (RR, 0.722; 95% CI, 0.609–0.857), although the Cox model did not reach statistical significance (HR, 0.857; 95% CI, 0.722–1.017; P = 0.077). Tirzepatide was associated with a significantly lower risk of deep vein thrombosis compared with semaglutide (RR, 0.703; 95% CI, 0.620–0.797; HR, 0.828; 95% CI, 0.730–0.939; P = 0.003). No statistically significant difference was observed for superficial vein thrombosis in the semaglutide comparator analysis (RR, 1.150; 95% CI, 0.753–1.757; HR, 1.346; 95% CI, 0.881–2.058; P = 0.168) (**Table 5**).

**Table 5:** Active comparator analysis: tirzepatide versus semaglutide.

Outcome	RR (95% CI)	HR (95% CI)	P value
Pulmonary embolism	0.722 (0.609–0.857)	0.857 (0.722–1.017)	0.077
Deep vein thrombosis	0.703 (0.620–0.797)	0.828 (0.730–0.939)	0.003
Superficial vein thrombosis	1.150 (0.753–1.757)	1.346 (0.881–2.058)	0.168

## 4 Discussion

In this large multicenter cohort study, initiation of tirzepatide was associated with a significantly lower 12-month risk of pulmonary embolism and deep vein thrombosis compared with propensity score-matched patients receiving lifestyle intervention alone, with no significant difference observed for superficial vein thrombosis. These associations remained consistent in a 90-day landmark sensitivity analysis, and active-comparator analyses against semaglutide showed a persistent reduction in deep vein thrombosis, with a lower cumulative risk of pulmonary embolism but no statistically significant difference by Cox regression. These findings suggest a potential association between tirzepatide use and lower observed rates of venous thromboembolic events in patients with type 2 diabetes and overweight or obesity. Although the observed associations were substantial, they should not be interpreted as evidence of a direct protective drug effect. Residual confounding, differential follow-up, and limitations inherent to electronic health record–based outcome ascertainment may contribute to the magnitude of the observed associations.

Venous thromboembolism is closely linked to metabolic disease, with obesity and diabetes promoting a prothrombotic state characterized by endothelial dysfunction, chronic low-grade inflammation, platelet activation, and impaired fibrinolysis (2). Weight reduction and improved glycemic control have been associated with partial reversal of these abnormalities, providing a biologically plausible framework through which metabolic therapies may influence thrombotic risk (9).

Being a dual glucose-dependent insulinotropic polypeptide (GIP) and glucagon-like peptide-1 (GLP-1) receptor agonist, tirzepatide produces substantial reductions in body weight (10). In clinical trial programs, tirzepatide demonstrated marked improvements in glycemic control and cardiometabolic risk factors, including blood pressure (11). More recently, in patients with obesity and obstructive sleep apnea, tirzepatide reduced hypoxic burden and systemic inflammation, further supporting a potential role in modulating vascular risk (12). The observed association with lower thromboembolic event rates, particularly for pulmonary embolism and deep vein thrombosis, is consistent with the hypothesis that systemic metabolic and inflammatory effects may contribute to differences in venous thrombotic risk.

Although incretin-based therapies have been extensively evaluated for their effects on atherosclerotic cardiovascular disease, their relationship with venous thromboembolism has been less clearly defined. Emerging observational data suggest that GLP-1 receptor agonists may be associated with a lower risk of venous thromboembolism, including pulmonary embolism, in patients with type 2 diabetes (7). Our findings add to this literature an under-studied area of specifically evaluating tirzepatide and demonstrating consistent reductions in both pulmonary embolism and deep vein thrombosis in a large, propensity-matched real-world cohort. Importantly, the association with deep vein thrombosis persisted in the active-comparator analysis against semaglutide, suggesting that the observed relationship may not be fully explained by treatment with incretin-based therapy alone. However, the pulmonary embolism finding was attenuated in the semaglutide comparator analysis, reaching statistical significance by cumulative risk analysis but not by Cox regression, supporting a more cautious interpretation.

The absence of a statistically significant association with superficial vein thrombosis may reflect differences in underlying pathophysiology. Superficial thrombophlebitis is frequently associated with local venous factors such as varicose veins, stasis, and endothelial inflammation, although systemic prothrombotic conditions may also contribute (13). In addition, the relatively low number of events may have limited statistical power to detect modest differences between groups. The absence of a significant superficial vein thrombosis signal in both the 90-day landmark and semaglutide comparator analyses further suggests that the observed associations were more consistent for deep venous thromboembolic outcomes (pulmonary embolism and deep vein thrombosis) than for superficial venous disease.

This study has several strengths, including its large sample size, multicenter design, and use of additional sensitivity analyses, active-comparator analyses, and incidence rate calculations accounting for differential follow-up. Propensity score matching improved balance across a wide range of demographic, clinical, and laboratory variables, and the consistency of findings across both risk-based and time-to-event analyses supports the robustness of the observed associations. In addition, reporting incidence rates per 1, 000 person-years helped account for differences in follow-up duration between cohorts and reduce overreliance on relative risk reduction alone.

Several limitations should also be considered when interpreting these findings. First, the observational nature of the study design inherently precludes firm conclusions regarding causality, as associations identified may be influenced by unmeasured or unknown factors. Second, although matching techniques were employed to improve baseline comparability between groups and reduce overt imbalances, the possibility of residual confounding remains, particularly from variables that were not captured or inadequately measured within the dataset. However, several VTE-relevant variables, including trauma, fracture history, immobility, central venous catheterization, COVID-19 infection status, and exact surgery timing, were unavailable or insufficiently granular within the TriNetX platform and may contribute to residual confounding. Although extensive propensity score matching was performed, residual confounding and unmeasured differences between cohorts may still partially explain the observed associations. Additionally, the lifestyle intervention cohort may differ from tirzepatide-treated patients in ways not fully captured within the available data, including healthcare-seeking behavior, access to specialist care, prescribing practices, treatment preferences, and socioeconomic factors. Such unmeasured differences may have contributed to the observed associations despite extensive matching. Third, study outcomes were ascertained using diagnostic and procedural codes rather than rigorously adjudicated clinical events, which introduces the potential for misclassification bias, either through coding inaccuracies or variability in clinical practice patterns. Additionally, outcome ascertainment relied on electronic health record diagnostic coding rather than independently adjudicated imaging-confirmed events. Consequently, imaging modality, venous segment localization, thrombus burden, and detailed thrombus chronicity beyond the coded diagnostic category were unavailable within the database structure. Fourth, although incidence rates per person-year and Cox regression were used to account for differential follow-up and censoring, mean follow-up duration still differed between cohorts, which may influence cumulative risk estimates. Fifth, the duration of follow-up was limited to 12 months, which may not be sufficient to capture longer-term outcomes, delayed effects, or the durability of observed associations over time. Moreover, BMI remained minimally imbalanced after matching (SMD 0.101), although the magnitude of imbalance was small. Finally, the TriNetX platform lacks granular longitudinal data on important clinical parameters such as changes in body weight, medication adherence, dose adjustments, and detailed glycemic trajectories, thereby constraining the ability to explore underlying mechanisms or fully contextualize the observed results. Additional sensitivity analyses such as negative-control outcomes and extensive subgroup analyses were not feasible within the scope of the current study and warrant future investigation.

## 5 Conclusion

In conclusion, in this large, propensity score-matched cohort derived from the TriNetX US Collaborative Network, initiation of tirzepatide was associated with significantly lower risks of pulmonary embolism and deep vein thrombosis compared with lifestyle intervention alone, while no statistically significant difference was observed for superficial vein thrombosis. These findings remained generally consistent in a 90-day landmark sensitivity analysis and were further supported by active-comparator analyses against semaglutide, particularly for deep vein thrombosis. These findings suggest a potential association between tirzepatide use and lower observed rates of venous thromboembolism in patients with type 2 diabetes and overweight or obesity. Although the observational design precludes causal inference, the consistency of the observed associations across thromboembolic outcomes and sensitivity analyses supports the hypothesis that improvements in weight, glycemic control, and systemic inflammation may contribute to reduced thrombotic risk. Clinically, these results may inform therapeutic decision-making in patients at elevated vascular risk, where treatment selection often considers both metabolic efficacy and cardiovascular safety. The persistence of lower incidence rates after accounting for person-time follow-up provides additional support for the observed association, while remaining non-causal. Future prospective studies and randomized trials are warranted to confirm these findings, elucidate underlying mechanisms, and determine whether this association translates into long-term reductions in thromboembolic morbidity and mortality.

## Data Availability

The datasets presented in this article are not readily available because the datasets analyzed in this study are subject to restrictions due to the use of the TriNetX Research Network platform and institutional data-use agreements. De-identified patient-level data are not publicly available and cannot be shared directly by the authors. Access to the data may be obtained through TriNetX by qualified institutions with appropriate permissions and licenses. Requests to access the datasets should be directed to the authors and through the TriNetX Research Network platform (https://trinetx.com/), subject to institutional approvals and applicable data-use agreements.
