# Supplementary material for: Tirzepatide and reduced risk of pulmonary embolism and deep vein thrombosis: a multicenter U.S. cohort study
Source: Front Endocrinol (Lausanne). 2026 Jul 1;17:1885961. doi: 10.3389/fendo.2026.1885961 (PMC13368568; doi:10.3389/fendo.2026.1885961)
Supplement: Supplementary file 1 [file DataSheet1.doc]

**STROBE Statement**

|  | Item No |  |
| --- | --- | --- |
| **Title and abstract** | 1 | (*a*) Tirzepatide and Reduced Risk of Pulmonary Embolism and Deep Vein Thrombosis: A Multicenter U.S. Cohort Study |
|  |  | (*b*) **Background** Tirzepatide is a dual glucose-dependent insulinotropic polypeptide and glucagon-like peptide-1 receptor agonist used in patients with type 2 diabetes and obesity. Although tirzepatide improves metabolic and cardiovascular risk factors, its association with venous thromboembolism, including pulmonary embolism and deep vein thrombosis, remains insufficiently characterized in real-world populations.  **Methods** We conducted a population-based retrospective cohort study using the TriNetX US Collaborative Network. Adults with type 2 diabetes and overweight or obesity who initiated tirzepatide were propensity score matched to patients receiving lifestyle intervention alone with no exposure to weight-loss medications. Outcomes included incident pulmonary embolism, deep vein thrombosis, and superficial vein thrombosis occurring between 30 days and 12 months after index. Additional analyses included a 90-day landmark sensitivity analysis and an active comparator analysis comparing tirzepatide with semaglutide.  **Results** After propensity score matching for demographic, metabolic, and clinical covariates, 235,200 patients were included in the primary analysis (117,600 per cohort). Tirzepatide use was associated with a significantly lower 12-month risk of pulmonary embolism compared with lifestyle intervention alone (RR, 0.215; 95% CI, 0.185–0.250; HR, 0.258; 95% CI, 0.222–0.299; log-rank P <0.001). Similar reductions were observed for deep vein thrombosis (RR, 0.303; 95% CI, 0.270–0.340; HR, 0.361; 95% CI, 0.322–0.406; log-rank P <0.001). No statistically significant difference was observed for superficial vein thrombosis (RR, 0.716; 95% CI, 0.484–1.060; HR, 0.868; 95% CI, 0.586–1.286; log-rank P =0.480). Findings for pulmonary embolism and deep vein thrombosis remained significant in the 90-day landmark analysis. In the semaglutide comparator analysis, tirzepatide was associated with a significantly lower risk of deep vein thrombosis, while the pulmonary embolism association was significant by risk-ratio analysis but not by Cox regression.  **Conclusions** In this large multicenter cohort study, tirzepatide use was associated with a lower risk of pulmonary embolism and deep vein thrombosis compared with lifestyle intervention alone. These findings suggest a potential association between tirzepatide use and lower observed rates of thromboembolic events in patients with diabetes and obesity. |
| Introduction | | |
| Background/rationale | 2 | VTE risk is elevated in T2DM/obesity; incretin therapies may modify thrombotic risk; limited evidence for tirzepatide. |
| Objectives | 3 | To evaluate association between tirzepatide and risk of PE, DVT, SVT compared with lifestyle intervention. |
| Methods | | |
| Study design | 4 | Retrospective cohort study using TriNetX. |
| Setting | 5 | TriNetX US network; follow-up 30–365 days. |
| Participants | 6 | Adults with T2DM and BMI ≥27; tirzepatide vs lifestyle; exclusions applied. |
|  |  | 1:1 propensity score matching (117,600 per cohort). |
| Variables | 7 | Defined exposure, outcomes (PE, DVT, SVT), and covariates. |
| Data sources/ measurement | 8* | Data from EHR codes (ICD-10, CPT, HCPCS, RxNorm). |
| Bias | 9 | PSM, landmark analysis, exclusion of prior outcomes. |
| Study size | 10 | Sample size based on eligible TriNetX population. |
| Quantitative variables | 11 | Continuous variables summarized; categorized for matching. |
| Statistical methods | 12 | RR, OR, KM analysis, Cox models; censoring applied. |
|  |  |  |
|  |  |  |
|  |  |  |
|  |  |  |

Continued on next page

| Results | | |
| --- | --- | --- |
| Participants | 13* | Pre-match: 128,618 vs 308,895 ; post-match: 117,600 each. |
|  |
|  |
| Descriptive data | 14* | Baseline balanced; follow-up ~255 vs 323 days. |
|  |
|  |
| Outcome data | 15* | *PE: 207 vs 960 ; DVT: 381 vs 1,249 ; SVT: 43 vs 60 .* |
|  |
|  |
| Main results | 16 | PE RR 0.215 ; DVT RR 0.303 ; SVT not significant. |
|  |
|  |
| Other analyses | 17 | Landmark analysis confirmed findings. |
| Discussion | | |
| Key results | 18 | Tirzepatide associated with reduced PE and DVT risk. |
| Limitations | 19 | Limitations: observational, coding, residual confounding. |
| Interpretation | 20 | Suggests protective association. |
| Generalisability | 21 | Generalizable to US T2DM + obesity population. |
| Other information | | |
| Funding | 22 | Institutional support from AUB; no role in study design or reporting. |

*Give information separately for cases and controls in case-control studies and, if applicable, for exposed and unexposed groups in cohort and cross-sectional studies.

**Note:** An Explanation and Elaboration article discusses each checklist item and gives methodological background and published examples of transparent reporting. The STROBE checklist is best used in conjunction with this article (freely available on the Web sites of PLoS Medicine at http://www.plosmedicine.org/, Annals of Internal Medicine at http://www.annals.org/, and Epidemiology at http://www.epidem.com/). Information on the STROBE Initiative is available at www.strobe-statement.org.
