# Supplementary material for: Tirzepatide and reduced risk of pulmonary embolism and deep vein thrombosis: a multicenter U.S. cohort study
Source: Front Endocrinol (Lausanne). 2026 Jul 1;17:1885961. doi: 10.3389/fendo.2026.1885961 (PMC13368568; doi:10.3389/fendo.2026.1885961)
Supplement: Supplementary file 2 [file DataSheet2.pdf]

Supplementary Table 1. Codes for Cohort Selection, Covariates, Outcomes, and Additional Analyses

Codes and terms are reported as represented in the TriNetX exports and revised manuscript. Laboratory and curated variables are listed using TriNetX identifiers when provided by the export.

A. Cohort Inclusion Criteria

Category	Code / Definition	Use in Analysis
Type 2 diabetes mellitus	ICD-10-CM: E11	Required inclusion criterion for all cohorts.
Overweight or obesity	ICD-10-CM: E66, or BMI at least 27 kg/m2 using TNX:9083	Required inclusion criterion for all cohorts.
Adult patients	Age at index; no diagnosis code used	Population restricted to adults in the study design.

B. Exposure and Comparator Definitions

Category	Code / Definition	Notes
Tirzepatide exposure	RxNorm: 2601723	Index exposure in the primary and active-comparator analyses.
Lifestyle intervention comparator	Dietary counseling: ICD-10-CM Z71.3<br>Exercise counseling: ICD-10-CM Z71.82<br>Face-to-face behavioral counseling for obesity: HCPCS G0447<br>Medical nutrition therapy: CPT 1013548	Comparator in the primary tirzepatide vs lifestyle intervention analysis.
Semaglutide active comparator	RxNorm: 1991302	Comparator in the active-comparator sensitivity analysis.

C. Medication Exclusions Used to Define No Prior Weight-Loss Medication Exposure

Medication	Code	Applied To
Phentermine	RxNorm: 8152	Tirzepatide, lifestyle intervention, and semaglutide comparator definitions as applicable.
Semaglutide	RxNorm: 1991302	Excluded from tirzepatide and lifestyle intervention cohorts; used as exposure in active-comparator analysis.
Liraglutide	RxNorm: 475968	Weight-loss medication exclusion.
Setmelanotide	RxNorm: 2469247	Weight-loss medication exclusion.
Orlistat	RxNorm: 37925	Weight-loss medication exclusion.
Naltrexone	RxNorm: 7243	Weight-loss medication exclusion.
Bupropion	RxNorm: 42347	Weight-loss medication exclusion.
Tirzepatide	RxNorm: 2601723	Excluded from lifestyle intervention and semaglutide comparator cohorts; used as exposure in tirzepatide cohorts.

D. Other Exclusion Criteria and Outcome-Specific Prior Event Exclusions

Category	Code / Definition	Notes
Prior pulmonary embolism	ICD-10-CM: I26	Used in cohort definitions and outcome-specific exclusion procedures.
Prior deep vein thrombosis	ICD-10-CM: I82.4	Outcome-specific exclusion before DVT analysis.
Prior superficial vein thrombosis	ICD-10-CM: I80.0	Outcome-specific exclusion before superficial vein thrombosis analysis.

Category	Code / Definition	Notes
Prior bariatric surgery	CPT: 43775	Excluded from all study cohorts.
Previous lifestyle intervention	HCPCS G0447; ICD-10-CM Z71.3; CPT 1013548; ICD-10-CM Z71.82	Excluded when defining the lifestyle-intervention comparator index event.

E. Outcome Definitions

Outcome	Code	TriNetX Outcome Definition
Pulmonary embolism	ICD-10-CM: I26	Pulmonary embolism.
Deep vein thrombosis	ICD-10-CM: I82.4	Acute embolism and thrombosis of deep veins of lower extremity.
Superficial vein thrombosis	ICD-10-CM: I80.0	Phlebitis and thrombophlebitis of superficial vessels of lower extremities.

F. Propensity Score Matching Covariates

Domain	Variable	Code / Identifier	Use
Demographics	Age at index	TriNetX age at index variable (AI)	Propensity score matching covariate.
Demographics	Sex / gender	Female (F), Male (M), Unknown gender (UN)	Propensity score matching covariate.
Demographics	Race	White: 2106-3; Black or African American: 2054-5; Asian: 2028-9; Unknown race: UNK	Propensity score matching covariate.
Demographics	Ethnicity	Not Hispanic or Latino: 2186-5; Hispanic or Latino: 2135-2; Unknown ethnicity: UN	Propensity score matching covariate.
Diagnosis	Essential hypertension	ICD-10-CM: I10	Propensity score matching covariate.
Diagnosis	Long-term current insulin use	ICD-10-CM: Z79.4	Propensity score matching covariate.
Diagnosis	Atherosclerotic heart disease of native coronary artery	ICD-10-CM: I25.1	Propensity score matching covariate.
Diagnosis	Pure hypercholesterolemia	ICD-10-CM: E78.0	Propensity score matching covariate.
Diagnosis	Disorders of lipoprotein metabolism and other lipidemias	ICD-10-CM: E78	Propensity score matching covariate.
Diagnosis	Neoplasms of unspecified behavior	ICD-10-CM: D49	Propensity score matching covariate.
Diagnosis	Ischemic heart diseases	ICD-10-CM: I20-I25	Propensity score matching covariate.
Diagnosis	Chronic kidney disease	ICD-10-CM: N18	Propensity score matching covariate.
Diagnosis	Nicotine dependence	ICD-10-CM: F17	Propensity score matching covariate.
Diagnosis	Long-term current use of anticoagulants and antithrombotics/antiplatelets	ICD-10-CM: Z79.0	VTE-relevant propensity score matching covariate.
Diagnosis	Cerebral infarction	ICD-10-CM: I63	Propensity score matching covariate.
Diagnosis	Injury of unspecified body region	ICD-10-CM: T14-T14	Propensity score matching covariate.
Diagnosis	Varicose veins of lower extremities	ICD-10-CM: I83	VTE-relevant propensity score matching covariate.
Diagnosis	Primary thrombophilia	ICD-10-CM: D68.5	VTE-relevant propensity score matching covariate.
Diagnosis	Hormone replacement therapy	ICD-10-CM: Z79.890	VTE-relevant propensity score matching covariate.
Diagnosis	Heart failure	ICD-10-CM: I50	Propensity score matching covariate.
Diagnosis	Obstructive sleep apnea	ICD-10-CM: G47.33	Propensity score matching covariate.
Procedure	Surgery	TriNetX curated procedure code: 1003143	Propensity score matching covariate.
Procedure	Hospital inpatient and observation care services	TriNetX curated procedure code: 1013659	Healthcare-utilization matching covariate.
Procedure	Pregnancy	ICD-10-PCS / TriNetX procedure category: 10	VTE-relevant propensity score matching covariate.
Procedure	Hematology and coagulation procedures	TriNetX curated procedure code: 1011759	Propensity score matching covariate.
Procedure	Chemotherapy	TriNetX curated procedure code: 1002	Cancer-treatment matching covariate.
Medication	Systemic contraceptives	TriNetX medication category: HS200	VTE-relevant propensity score matching covariate.

Domain	Variable	Code / Identifier	Use
Laboratory	BMI	TNX:9083	Propensity score matching covariate and obesity-related inclusion variable.
Laboratory	Hemoglobin A1c / hemoglobin.total in blood	TNX:9037	Propensity score matching covariate.
Laboratory	Cholesterol [mass/volume] in serum or plasma	TNX:9000	Propensity score matching covariate.
Laboratory	SARS coronavirus 2 and related RNA [presence]	TNX:9088	Propensity score matching covariate.

G. Analysis Windows and Sensitivity Analyses

Analysis	Time Window / Definition	Comparator	Notes
Primary analysis	Outcomes assessed from 30 days through 365 days after index, as specified in the manuscript.	Tirzepatide vs lifestyle intervention	Primary cohort comparison.
90-day landmark sensitivity analysis	Outcome assessment begins 90 days after index and continues through 365 days.	Tirzepatide vs lifestyle intervention	Used to reduce early surveillance and protopathic bias.
Active-comparator analysis	Same outcome definitions and follow-up framework as the primary analysis.	Tirzepatide vs semaglutide	Semaglutide defined using RxNorm 1991302.

Abbreviations: BMI, body mass index; CPT, Current Procedural Terminology; HCPCS, Healthcare Common Procedure Coding System; ICD-10-CM, International Classification of Diseases, Tenth Revision, Clinical Modification; RxNorm, normalized medication nomenclature; TNX, TriNetX curated/laboratory identifier; VTE, venous thromboembolism.

Note: Some reviewer-requested variables such as exact surgery timing, immobility, central venous catheterization, and detailed fracture timing were not available as sufficiently granular matching variables in the TriNetX export and are therefore not listed as matched covariates.
